# *Baylisascaris procyonis* Roundworm in Common Raccoon (*Procyon lotor*), Mexico

**DOI:** 10.3201/eid3106.241672

**Published:** 2025-06

**Authors:** Ana Luisa Gómez-Sánchez, Carlos Daniel Pinacho-Pinacho, Juan José Barrios-Gutierrez, Ismael Guzmán-Valdivieso, Alberto Gonzalez-Romero, Tania Fonseca-Leal, Andrés M. López-Pérez

**Affiliations:** Instituto de Ecología-INECOL A.C., Xalapa, Mexico

**Keywords:** *Baylisascaris procyonis*, roundworm, nematode, raccoon, *Procyon lotor*, parasites, zoonoses, Mexico

## Abstract

We found the zoonotic nematode, *Baylisascaris procyonis*, in a common raccoon (*Procyon lotor*) in Mexico. Expansion of raccoons into human-dominated regions might increase the risk of *B. procyonis* infections in humans. Increased surveillance and healthcare provider awareness of baylisascariasis in Mexico will be needed to prevent those infections in humans.

*Baylisascaris procyonis*, also known as the raccoon roundworm, is a zoonotic ascarid nematode that parasitizes the small intestine of its definitive host, the common raccoon (*Procyon lotor*). *B. procyonis* worms are commonly found in the United States and Canada (*1*). The *B. procyonis* life cycle involves direct transmission via the fecal–oral route between raccoons, but raccoons can also acquire the parasite indirectly by ingesting infected paratenic (transport) hosts (*1*,*2*). In the definitive host, the parasite life cycle occurs in the small intestine, where larvae hatch from infective eggs and develop into adults; male and female adult worms then mate and eggs are oviposited. However, larvae do not develop to adult stages in humans and other paratenic hosts. Instead, once the larvae hatch, they rapidly penetrate the intestinal mucosa and migrate through blood to the liver, lungs, or other tissues, causing visceral, ocular, or neural larval migrans, which can result in fatal disease or neurologic disease with severe outcomes (*1*,*2*). Exposure risk is highest for young children, who are more likely to accidentally ingest animal feces (*1*,*3*).

*B. procyonis* roundworms are considered a rare cause of human disease; ≈40 cases have been reported worldwide (*3*). However, human infection is increasingly recognized as an emerging public health concern in North America and in some countries in Europe and Asia, where raccoons have been introduced (*1*,*2*).

In January 2024, we captured a juvenile (<1 year of age), male raccoon in a patch of cloud forest in Veracruz, Mexico (latitude 19°30'47''N, longitude 96°56'33''W) that showed clinical signs consistent with distemper virus infection. Because of illness severity, we euthanized the raccoon and subsequently performed a necropsy. We conducted all handling procedures according to the Universidad Veracruzana Institutional Animal Care and Use Committee protocol (no. 003/2024-UV-IACUC) and the collection permit issued by the Mexico Secretary of Environment (permit no. SPARN/DGVS/06330/23). During necropsy, we collected 4 roundworms from the small intestine. We preserved 2 worms in 96% ethanol for molecular testing and fixed the other 2 worms in 4% formalin for morphologic identification. We did not detect eggs in the feces by using sedimentation or flotation tests.

All 4 roundworms were initially identified as *Baylisascaris* sp. by using a Leica DM750 light microscope (Leica, https://www.leica-microsystems.com) (*4*). To better characterize morphology, we processed 1 female specimen for scanning electron microscopy using standard methods, including washing, rinsing, and dehydrating steps, followed by a critical point drying step and mounting on aluminum stubs with a carbon adhesive before sputter coating with gold in a Quorum Q150RS instrument (Quorum Technologies, https://www.quorumtech.com). We produced scanning electron micrographs of the female specimen (Figure 1). We deposited 1 male *B. procyonis* roundworm specimen into the Colección Nacional de Helmintos del Instituto de Biología at the Universidad Nacional Autonoma de Mexico in México City (accession no. CNHE 8585).

**Figure 1:**
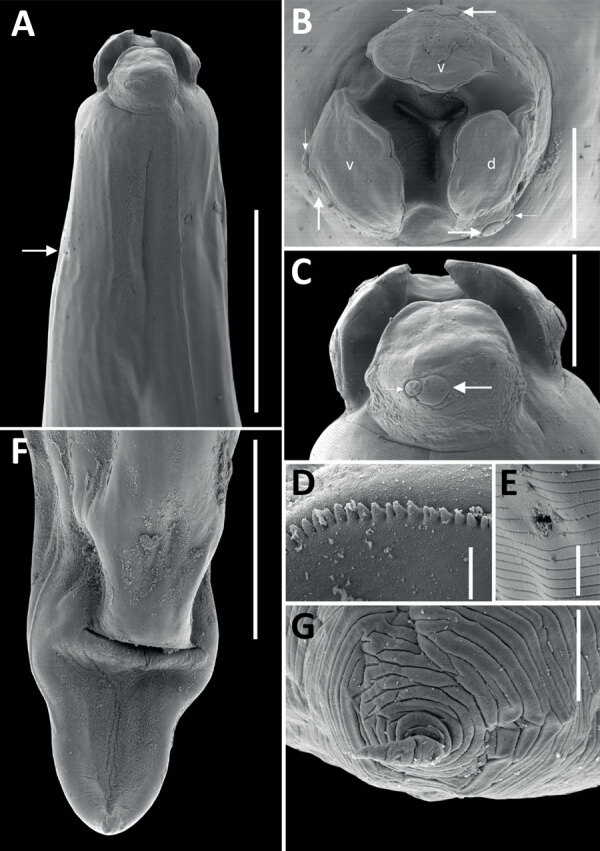
Scanning electron micrographs of female *Baylisascaris procyonis* roundworm detected in common raccoon (*Procyon lotor*), Mexico. A) Anterior end of worm body. Arrow indicates the excretory pore. Scale bar indicates 400 μm. B) Cephalic end, apical view of worm. Lowercase letters indicate dorsal (d) and ventral (v) lips, each having 1 large double papillae instead of a dorsal lip with 2 large double papillae, as reported previously (*4*). Small and large arrows indicate the double papillae. Scale bar indicates 100 μm. C) Cephalic end of worm. Small and large arrows indicate the double papillae. Scale bar indicates 100 μm. D) Magnified image of labial denticles. Scale bar indicates 5 μm. E) Magnified image of the excretory pore. Scale bar indicates 10 μm. F) Posterior end of the worm body. Scale bar indicates 300 μm. G) Magnified image of the tail tip. Scale bar indicates 10 μm.

We extracted genomic DNA from 2 worms by using DNAzol (Molecular Research Center, https://www.mrcgene.com) (*5*). To confirm morphologic identification of *Baylisascaris* sp., we amplified partial segments of the domains D2–D3 of the 28S rDNA gene (785 bp) by using 502 and 536 primers and conditions, as previously described (*6*). Macrogen Inc. (https://dna.macrogen.com) purified and sequenced the PCR samples. We assembled contigs and resolved base-calling differences by using Geneious version 8.1.8 (https://www.geneious.com). We generated consensus sequences and compared them with sequences in GenBank. Using MEGA11 (*7*), we aligned sequences by using the ClustalW algorithm and constructed phylogenetic trees by using the maximum-likelihood method. Phylogenetic analysis of the 28S rRNA sequences showed 100% identity with *B. procyonis* worms collected from raccoons in the United States (GenBank accession no. KP843605), China (accession no. OR457646) and Norway (accession no. KC543470) (Figure 2). We submitted sequences from this study to GenBank (accession nos. PQ471568 and PQ471569).

**Figure 2:**
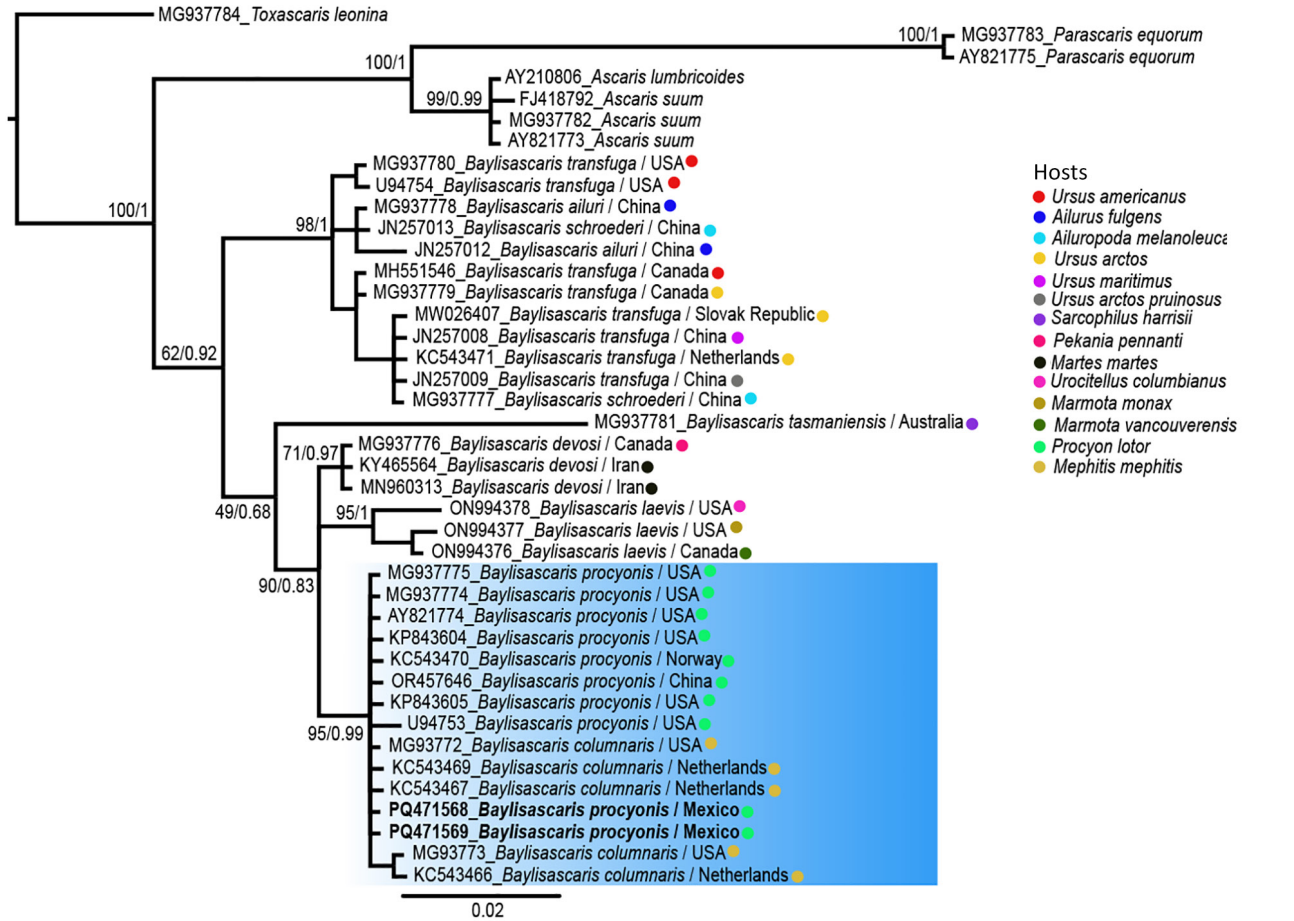
Phylogenetic analysis of *Baylisascaris procyonis* roundworms obtained from common raccoon (*Procyon lotor*), Mexico. Maximum-likelihood and Bayesian inference tree of *Baylisascaris* spp. according to the general time-reversible model with gamma distribution. Blue shading indicates GenBank sequences with greatest identity to sequences from Mexico (bold font). Numbers near internal nodes indicate bootstrap values/posterior probability of clade frequencies. Colored dots indicate host species. Scale bar indicates nucleotide substitutions per site.

Finding *B. procyonis* roundworms in a raccoon in Mexico extends the geographic distribution of this zoonotic nematode in the Americas, which has been described throughout Canada and the United States and, in 1 report, from Costa Rica (*1*,*8*). Despite the relatively low number of human cases reported worldwide and previous absence of *B. procyonis* worms in Mexico, the ecoepidemiology of *B. procyonis* roundworms suggests that the number of infection cases are underestimated. Infection prevalence is high in raccoons across their known distribution range; infected animals can carry several adult worms, which can excrete millions of eggs into the environment (*1*). A high infection prevalence likely also applies to Mexico because raccoons are distributed throughout all the states within the country, and they have increasingly become synanthropic, living close to human settlements, where they are also occasionally kept as pets (*9*,*10*). In addition, domestic dogs could also play a key role in the epidemiology of *B. procyonis* roundworms, and other zoonotic ascarids such as *Toxocara canis*, which raises the risk for human exposure and infection (*1*,*2*). Our findings highlight the importance of increasing epidemiologic surveillance and healthcare provider awareness of baylisascariasis in Mexico to effectively prevent infections, particularly in areas where humans (especially children) and dogs might come into contact with raccoons or *B. procyonis* eggs in the environment.
